# Development of Novel Arginase Inhibitors for Therapy of Endothelial Dysfunction

**DOI:** 10.3389/fimmu.2013.00278

**Published:** 2013-09-17

**Authors:** Jochen Steppan, Daniel Nyhan, Dan E. Berkowitz

**Affiliations:** ^1^Department of Anesthesiology and Critical Care Medicine, The Johns Hopkins Medical Institutions, Baltimore, MD, USA

**Keywords:** endothelium, endothelial dysfunction, arginase, l-arginine, nitric oxide synthase, reactive oxygen species, diabetes mellitus

## Abstract

Endothelial dysfunction and resulting vascular pathology have been identified as an early hallmark of multiple diseases, including diabetes mellitus. One of the major contributors to endothelial dysfunction is a decrease in nitric oxide (NO) bioavailability, impaired NO signaling, and an increase in the amount of reactive oxygen species (ROS). In the endothelium NO is produced by endothelial nitric oxide synthase (eNOS), for which l-arginine is a substrate. Arginase, an enzyme critical in the urea cycle also metabolizes l-arginine, thereby directly competing with eNOS for their common substrate and constraining its bioavailability for eNOS, thereby compromising NO production. Arginase expression and activity is upregulated in many cardiovascular diseases including ischemia reperfusion injury, hypertension, atherosclerosis, and diabetes mellitus. More importantly, since the 1990s, specific arginase inhibitors such as *N*-hydroxy-guanidinium or *N*-hydroxy-nor-l-arginine, and boronic acid derivatives, such as, 2(*S*)-amino-6-boronohexanoic acid, and *S*-(2-boronoethyl)-l-cysteine, that can bridge the binuclear manganese cluster of arginase have been developed. These highly potent and specific inhibitors can now be used to probe arginase function and thereby modulate the redox milieu of the cell by changing the balance between NO and ROS. Inspired by this success, drug discovery programs have recently led to the identification of α–α-disubstituted amino acid based arginase inhibitors [such as (*R*)-2-amino-6-borono-2-(2-(piperidin-1-yl)ethyl)hexanoic acid], that are currently under early investigation as therapeutics. Finally, some investigators concentrate on identification of plant derived compounds with arginase inhibitory capability, such as piceatannol-3′-O-β-d-glucopyranoside (PG). All of these synthesized or naturally derived small molecules may represent novel therapeutics for vascular disease particularly that associated with diabetes.

## Nitric Oxide and Its Regulation by Arginase

The endothelium plays a major role in cardiovascular physiology. The intact structure and integrity is vital for endothelial cells in order to fulfill their role separating blood flow from surrounding tissues and ensuring an anti-thrombogenic surface. Previously only known of as a passive barrier between those two, the endothelium is now considered a main hub for regulating vascular tone, hemostasis, immune function, structure, smooth muscle cell proliferation, and migration. The combined amount of surface area of the endothelium can reach up to 350 m^2^ in total (1). Endothelial dysfunction has been identified as an early harbinger of multiple diseases and resulting vascular pathology. One of the major contributors to endothelial dysfunction is a decrease in nitric oxide (NO) bioavailability, impaired NO signaling, and an increase in the amount of reactive oxygen species (ROS). NO is not only a potent vasodilator and essential in regulating vascular tone and blood pressure, but it also contributes to the regulation of hemostasis, platelet, and leukocyte adhesion as well as vascular smooth muscle cell proliferation. It is freely diffusible with a half-life of just a few seconds prior to its conversion into nitrates and nitrites that are ultimately excreted. NO is synthesized by nitric oxide synthase (NOS), a family of P450 mono-oxygenase-like enzymes which exist in one of three isoforms: nNOS or NOS-1 (neuronal NOS in the central nervous system, skeletal muscle, and pancreas), iNOS or NOS-2 (inducible NOS in activated macrophages, heart, liver, and smooth muscle cells), and eNOS or NOS-3 (endothelial NOS in the endothelium, brain, and epithelium). In the endothelium NO is produced by eNOS (endothelial NOS), which uses l-arginine as a substrate after activation by either chemical agonists or mechanical forces (shear stress). The process of NO synthesis involves firstly the oxidation of arginine to NG-hydroxy-l-arginine (NHA) using nicotinamide adenine dinucleotide phosphate (NADPH) and O_2_ catalyzed by NOS (2). The second step involves the production of NO when NHA is converted to l-citrulline via NOS. The actions of NOS are accelerated by the cofactors flavin adenine dinucleotide (FAD), flavin mononucleotide (FMN), and tetrahydrobiopterin (BH4). In the absence of its substrate l-arginine or its cofactor BH4, eNOS uncouples and produces ROS, making it one of the four major enzymes involved in the production of vascular ROS. (The others are xanthine oxidase, NADH/NADPH, the mitochondrial electron transport chain, and eNOS). NOS uncoupling is an important contributor to endothelial dysfunction and plays a crucial role in the cardiovascular phenotype. Arginase, a critical urea cycle enzyme, also utilizes l-arginine. It thereby directly competes with eNOS for their common substrate l-arginine and constrains its availability to eNOS, compromising NO production and increasing the production of ROS by NOS uncoupling (3– 5). Arginase, which is present in two isoforms (arginase I in the liver and arginase II extrahepatic) catalyzes the final step of the urea cycle yielding l-ornithine and urea from l-arginine. Arginase II appears to be the predominant isoform in human endothelial cells (6) and is highly compartmentalized. There appear to be at least three distinct pools of l-arginine that are spatially confined and regulated by different transporters and enzymes (7, 8). Thus, local concentrations of l-arginine in microdomains in which NOS and/or arginase might be located may be limiting for NOS isoforms. This concept of the l-arginine paradox is found in the mammalian organism by which l-arginine concentrations by far exceed *K*_m_ values of NOS. Consequently, additional l-arginine should not augment nitric oxide formation. *In vivo* however, increasing the plasma concentration of l-arginine has repeatedly been shown to increase NO production (4). The three existing pools of arginine within the cell are (1) a freely exchangeable pool (pool I) with extracellular l-arginine that is regulated by the cationic transporter (CAT-1) and depleted by exchanging the pool with cationic amino acid lysine, (2) a non-freely exchangeable pool (pool II) with extracellular l-arginine that cannot depleted by l-lysine, and (3) extracellular l-arginine pools (pool III) present in endothelial cells and mitochondria in which arginase II modulates NO synthesis through a non-freely exchangeable l-arginine pool (9). According to recent paradigms, the not freely exchangeable l-arginine pool II is composed of two cytosolic microdomains. The major function of pool IIA appears to be the result of citrulline recycling and conversion to arginine by a combined reaction of argininosuccinate synthetase and argininosuccinate lyase (10). The remaining l-arginine pool IIB, which is mainly used by mitochondria, is composed of l-arginine gained by protein breakdown and cannot be depleted by neutral amino acids such as histidine. Arginase expression and activity is upregulated in many diseases including ischemia reperfusion injury (in the heart, lung, and kidneys), hypertension, atherosclerosis, aging, diabetes mellitus, erectile dysfunction, pulmonary hypertension, and aging. Furthermore it can be induced by lipopolysaccharide (LPS), TNFα, interferon γ, 8-bromo-cGMP, and hypoxia (11–14). It has been shown repeatedly that both arginase isoforms are capable of reciprocally regulating NO production (3, 4, 15). More importantly the development of specific arginase inhibitors like *N*-hydroxy-guanidinium or boronic acid derivatives, such 2(*S*)-amino-6-boronohexanoic acid, and *S*-(2-boronoethyl)-l-cysteine (BEC) can now be used to probe arginase function (16). This development in the 1990s allowed the selective inhibition of arginase in the laboratory and thereby the modulation of the substrate availability for NOS and its end product NO (17–19).

## Arginase Structure, Enzymatic Function, and Inhibitor Design

**Figure 1:**
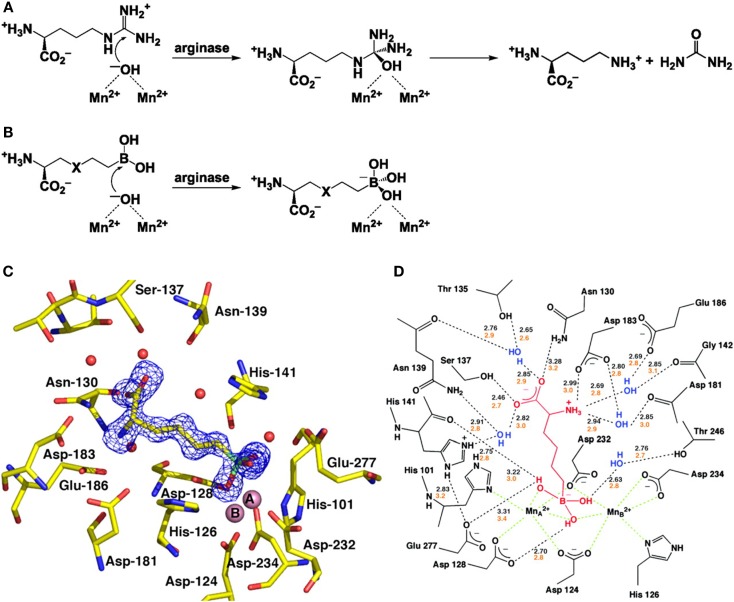
**Structure and function of arginase and the interaction with BEC**. **(A)** The formation of L-ornithine and urea from l-arginine by arginase. **(B)** The reaction of the boronic acid analogs of l-arginine, 2(*S*)-amino-6-hexanoic acid (ABH) (X representing CH2) and *S*-(2-boronoethyl)-l-cysteine (BEC) (X representing S). **(C)** Electron density map of ABH bound to human arginase I. **(D)** A schematic showing the enzyme-inhibitor hydrogen bond (black dashed lines) and metal coordination interactions (green dashed lines). With kind permission from Santhanam et al. (55).

The first step toward the generation of arginase inhibitors was the determination of the crystal structure of arginase and its active site. Dr. Christianson and his laboratory team from the University of Pennsylvania first demonstrated the binuclear manganese cluster required for catalysis at the active side of rat arginase using X-ray crystallography (20). Successive studies determined the structures of human arginase I (21) and human arginase II (22), both of which contain almost identical metal clusters and active site configurations, this similarity makes it very difficult to develop inhibitors that are specific for one arginase isoform. At the active site, l-ornithine and urea are formed by the collapse of a tetrahedral intermediate that forms after the addition of a hydroxide ion to the l-arginine guanidinium group in the binuclear manganese cluster (Figures 1A,B).

The first group of arginase inhibitors consisted of the boronic acid analogs of l-arginine (2)*S*-amino-6-hexanoic acid (ABH) and *S*-2-BEC both of which inhibit the catalytic activity of arginase (16, 23, 24). As both contain trigonal planar boronic acid moieties instead of a trigonal planar guanidinium group, found in l-arginine, binding to the active site of arginase results in a nucleophilic attack of the boron atoms by the metal-bridging ion, resulting in a tetrahedral boronate ion (18). This reaction is identical to the creation of a tetrahedral intermediate by nucleophilic attack of hydroxide ions at the guanidinium group of l-arginine and has been confirmed by crystallographic structure determination (18, 22, 24) (Figures 1C,D). The ability of the boronic side chains of ABH and BEC to bind the active side chain of arginase is 50,000 times stronger than the binding of comparable amino acids, aldehyde, or tetrahedral sulfonamide, both of which mimic the tetrahedral intermediate in the arginase mechanism (22, 25). ABH [*K*_i_ = 0.11 μM for arginase I and *K*_i_ = 0.25 μM (at pH of 7.5) for arginase II (26, 27)] and BEC [*K*_i_ = 0.4–0.6 μM for arginase I and *K*_i_ = 0.31 μM (at pH of 7.5) for arginase II (18)] are therefore specific inhibitors of arginase as they are closely matched to the metal-bridging hydroxide ion in the active site of arginase.

Another category of arginase inhibitors, that is mainly represented by *N*-hydroxy-l-arginine (NOHA) and *N*-hydroxy-nor-l-arginine (nor-NOHA), is characterized by *N*-hydroxy-guanidinium side chains (25, 28–30). Analysis of the enzyme structure by X-ray crystallography reveals that both NOHA and nor-NOHA inhibit arginase by displacing the metal-bridging hydroxide ion of arginase with their *N*-hydroxy group (31). Based on this mechanism, both amino acids inhibit arginase activity with nor-NOHA being a more potent inhibitor (*K*_i_ = 500 mM for nor-NOHA vs. *K*_i_ = 10 μM for NOHA) (28, 30) and with both being less specific than the boronic acid derivates BEC and ABH [for nor-NOHA the *K*_i_ values for arginase I and arginase II are 500 and 50 nM, respectively (32)].

**Figure 2:**
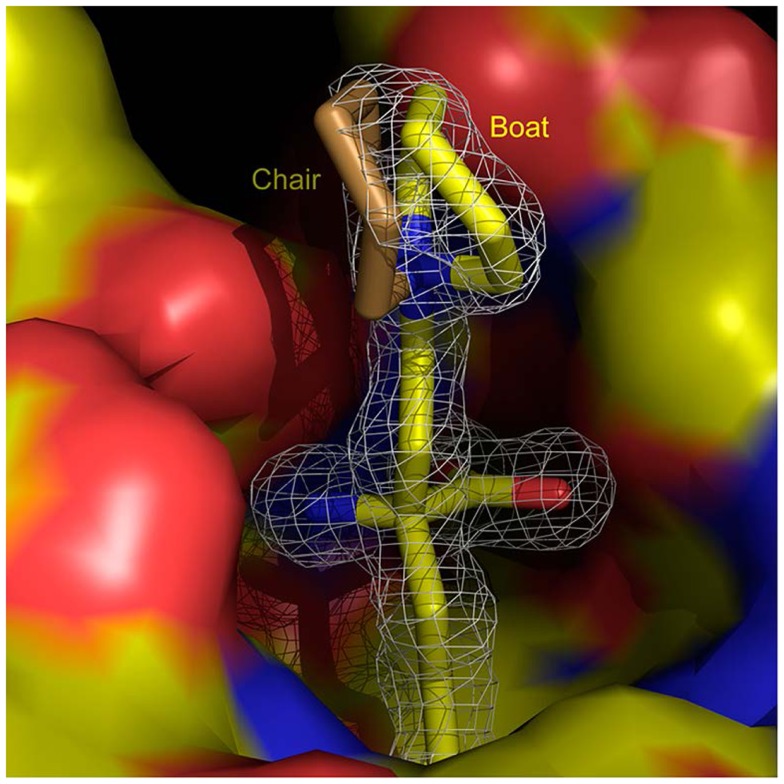
**(*R*)-2-amino-6-borono-2-(2-(piperidin-1-yl)ethyl)hexanoic acid) and arginase 1**. Structure of (*R*)-2-amino-6-borono-2-(2-(piperidin-1-yl)ethyl)hexanoic acid) at the active site of arginase I (surface colored according to charge), shown superimposed with a difference map calculated without the inhibitor model contoured at 3 RMS (white contours). With kind permission from Ref. (33).

Recent efforts now concentrate on expanding the range of arginase inhibitors based on a structure based design program, translating ABH’s mechanism of action into new compounds (33, 34). Identifying the α-position of ABH as a target for site substitution, a tertiary amine linked via a two-carbon chain improves the ability of ABH to inhibit both arginase I and arginase II (35). X-ray crystallography demonstrates a close contact between nitrogen and the carboxylic side chain of Asp 181 (arginase I) and Asp 200 (arginase II) at the active site (Figure 2) (35). This has led to the discovery of (*R*)-2-amino-6-borono-2-(2-(piperidin-1-yl)ethyl)hexanoic acid (compound 9) a small molecule that has shown efficacy in the attenuation of myocardial reperfusion injury (33). Compound 9 contains a piperidine linked to the α-carbon by a two-carbon aliphatic chain at the α-position. This results in the formation of new through-water hydrogen bonding interaction with Asp 181 and Asp 183 (arginase I), providing a roughly sixfold increase in potency compared to ABH. Co-crystallizing compound 9 with arginase II yields a similar, albeit weaker interaction of the through-water contacts between the piperidine ring nitrogen atom and Asp 200 and Asp 202 (arginase II: IC50 509 nM vs. arginase I: IC50 223 nM).

In a parallel approach to finding new and improved inhibitors of arginase, some investigators have concentrated on characterizing a plant derived compound with the ability to inhibit arginase (36, 37). It has been demonstrated that piceatannol-3′-*O*-β-d-glucopyranoside (PG), an important component of rhubarb extract has, antioxidant effects (38), Woo et al. tested the ability of this extract to act as an arginase inhibitor (36). They were able to demonstrate that PG inhibits arginase I and arginase II activity and increased nitric oxide production in a dose-dependent manner. In their experiments PG proved to be a non-specific arginase inhibitor with an IC50 value of 11.22 μM (arginase I) and 11.06 μM (arginase II) respectively (36). Furthermore they were able to extend their studies by demonstrating that PG improves endothelial dysfunction via eNOS activation in a rodent model of hyperlipidemia (39).

This search for a new plant derived arginase II specific inhibitor has very recently been extended by screening hundreds of plant extracts for potential targets (37). This investigation yielded a methanol extract of *Scutellaria indica*, that has the ability inhibit arginase II. Following multiple additional fractionations, and repeated column chromatography, the group was able to isolate eight different compounds from the extract. One of the compounds (compound 1, flavan type) has been previously unknown while the remaining seven compounds (compound 2–8) have been described earlier. Arginase II activity was inhibited by two of the eight compounds (compound number 3 and 5) with an IC50 of 25.1 and 11.6 μM, respectively (37). They authors did not test the capability of the extract or compounds to inhibit arginase I, nor did they investigate the underlying mechanism of arginase II inhibition.

## Implications of Inhibitors in Diabetes Mellitus

Diabetes mellitus has long been shown to be a disease tightly associated with endothelial dysfunction (40). Recent data suggests that metabolic degradation of l-arginine is directly involved in the l-arginine enhanced insulin-stimulated glycogen synthesis (41). Furthermore, hyperglycemia and the hemoglobin A1C levels correlate to arginase activity, with arginase activity being increased in type 2 diabetic subjects with impaired NOS activity (42–44). These higher arginase activity levels could be a result of reduced insulin action and increased protein catabolic processes in diabetic subjects (45). Consequently, insulin treatment reverses increased arginase activity and mRNA levels to close to control values (46). In addition to the effect of arginase in the endothelium of diabetic patients, arginase is also present and active in human islets cells of the pancreas, where arginase activity regulates the generation of NO (47).

The molecular mechanism of glucose-induced upregulation of arginase activity appears to involve small G proteins. In fact, the Rho kinase inhibitor Y-27632 as well as a HMG-coenzyme reductase inhibitor (statin) blunt the upregulation of the enzyme as well as ROS production under these conditions. Therefore, statins, which are known to inhibit the Rho/Rock pathway, reduce vascular events in patients with diabetes in part by a mechanism that involves inhibition of arginase activation (48, 49). Moreover, studies show that diabetes-induced impairment of vasorelaxation is correlated with increases in ROS, arginase activity, and arginase expression in the aorta. A treatment regime with simvastatin or l-citrulline is able to blunt these effects and acute treatment of diabetic coronary arteries with arginase inhibitors has been shown to reverse the impaired vasodilation to acetylcholine (50). This is likely due to the upregulation of arginase I in coronary arterioles of diabetic patients, which contributes to reduced NO production and consequently diminished vasodilation (51). Thus, endothelial dysfunction in diabetes may be caused, at least partially, by reduced l-arginine availability for eNOS. Given both preclinical data from animal models, early but provocative human data, as well as potent small molecule inhibitor drug candidates, arginase promises to be an exciting, novel target for therapy in diabetic vasculopathy, a scourge for which there is currently little effective treatment. However caution is advised in selectively inhibiting arginase isoforms in macrophages. The inflammatory phenotype M1 macrophages (Th1 immune response) mainly expresses arginase II, while the profibrotic and repair phenotype M2 (alternatively activated macrophage, Th2 cytokine response) mainly expresses arginase I (52). Therefore selective inhibition of arginase I might lead to an expansion of the M1 phenotype, which could aggravate iNOS mediated inflammatory effects (53), while selective arginase II inhibition might enhance the profibrotic response of alternatively activated ornithine producing macrophages with potential deleterious effects on vessels and other organs (54).

## Conflict of Interest Statement

The authors declare that the research was conducted in the absence of any commercial or financial relationships that could be construed as a potential conflict of interest.
